# Diabetes-Mediated STEAP4 Enhances Retinal Oxidative Stress and Impacts the Development of Diabetic Retinopathy

**DOI:** 10.3390/antiox14020205

**Published:** 2025-02-11

**Authors:** Brooklyn E. Taylor, Scott J. Howell, Chieh Lee, Zakary Taylor, Katherine Barber, Patricia R. Taylor

**Affiliations:** 1 Louis Stokes Cleveland VA Medical Center, Cleveland, OH 44106, USA; bxt202@case.edu (B.E.T.); sjh36@case.edu (S.J.H.); cxl77@case.edu (C.L.); kxb543@case.edu (K.B.); 2 Department of Ophthalmology and Vision Science, Case Western Reserve University, Cleveland, OH 44106, USA; zxt154@case.edu

**Keywords:** diabetic retinopathy, STEAP4, oxidative stress

## Abstract

Diabetic retinopathy is the most common diabetic complication of the microvasculature and one of the leading causes of acquired vision loss worldwide. Yet, the current treatments for this blinding disease are futile to many diabetics. Accordingly, new biomarkers and therapeutics for diabetic retinopathy are needed. We discovered that *STEAP4* (Six-Transmembrane Epithelial Antigen of the Prostate 4) is significantly increased in peripheral blood mononuclear cells of diabetics. *STEAP4* expression was gradiently increased from low levels in diabetics without retinopathy to successively higher levels in diabetics with more severe disease. Although the role of STEAP4 in the diabetic retina is unclear, these results provide strong evidence that this metabolic enzyme could be a potential biomarker for diabetic retinopathy progression. Thus, the central goal of this study was to evaluate if this potential biomarker impacts the intrinsic pathologies that lead to the development of diabetic retinopathy. In diabetic mice, STEAP4 was significantly increased and co-localized with 4-Hydroxy-2-nonenal in the Müller glia and photoreceptor layers of the retina. STEAP4 inhibition significantly decreased reactive oxygen species in murine photoreceptor cells, human Müller glia, and retinas of diabetic mice. Administering an intravitreal injection of anti-STEAP4 to diabetic mice halted Occludin degradation in the retinal vasculature. Similarly, anti-STEAP4 treatment of human retina endothelial cells halted cell death mediated by diabetic donor sera. Collectively, our findings provide strong evidence that STEAP4 impacts the intrinsic pathologies that initiate the development of diabetic retinopathy. Suggesting that STEAP4 could be a novel biomarker and clinically relevant therapeutic target for this diabetic complication and blinding disease.

## 1. Introduction

More than 10% of the US population is diabetic, while more than half suffer from (DR) diabetic retinopathy [1,2]. However, there is no treatment for non-proliferative DR (NPDR), there are no biomarkers for the progression of DR, and treatments for late-stage proliferative diabetic retinopathy (PDR) are futile to ~30% of the diabetics receiving care [3,4]. With such a significant healthcare issue, new biomarkers and therapeutic targets are required to halt the progression of this incurable microvascular disease.

The etiology of DR is multifactorial. Yet, multiple studies provide strong evidence that hyperglycemia enhances retinal oxidative stress, which leads to the development of DR [5,6,7,8,9]. One of the diabetes-mediated mechanisms of oxidative stress is Ferrous iron (Fe^2+^) accumulation in the retina [10]. Diabetes initiates Fe^2+^ uptake in the retina [10]. The retina’s compensatory mechanism for this iron overload is to oxidize the excess Fe^2+^ to elicit extracellular release of (Fe^3+^) ferric iron [11]. Through the Fenton reaction, ferrous iron is oxidized by hydrogen peroxide, which generates ferric iron and hydroxyl radicals [12]. These hydroxyl radicals are toxic reactive oxygen species that exacerbate oxidative stress in the diabetic retina. Enhanced oxidative stress causes retinal endothelial cell death and tight junction protein degradation in the retinal microvasculature. This can lead to retinal vascular leakage, capillary non-perfusion, and the development of NPDR and/or diabetic macular edema [13,14,15].

Per the literature, *Steap4* is upregulated in the retina of diabetic rats [16]. This is of interest because STEAP4 is a ferroxidase that catalyzes the reduction of extracellular Fe^3+^ to Fe^2+^ for cellular uptake [17,18]. Since STEAP3 maintains iron homeostasis in the thriving retina [19,20], it is unknown why only *Steap4* is upregulated in the diabetic retina. When extracellular Fe^3+^ binds to the C-terminal transmembrane domain of STEAP4, NADPH oxidoreductase binds to the cytosolic N-terminal domain of STEAP4 [21]. This induces extracellular iron reduction, intracellular iron uptake, and cellular production of reactive oxygen species (ROS). In this current study, we discovered that *STEAP4* is upregulated in the blood of diabetic patients and that levels of *STEAP4* correlate to the severity of DR. Suggesting that STEAP4 could be a clinically relevant biomarker for the progression of DR. Since STEAP4 can induce ROS production while catalyzing iron, we postulated that STEAP4 would enhance oxidative stress in the diabetic retina. Consequently, increased oxidative stress should impel vascular damage in the diabetic retina, which can lead to the onset of DR. Thus, we hypothesized that STEAP4 inhibition in the diabetic retina would decrease retinal oxidative stress and arrest vascular impairment. Thus, it is the overarching goal of this study to define the role of this potentially novel biomarker in the development of diabetic retinopathy.

## 2. Materials and Methods

### 2.1. Blood Collection and PBMC Sample Preparation from Non-Diabetic and Diabetic Patients

Non-diabetic and Type II diabetic male patients were enrolled in this IRB-approved clinical study at Louis Stokes Cleveland VA Medical Center during routine eye exams. Written patient consent was provided prior to blood and patient record collection. Non-diabetic patients and diabetic patients without diabetic retinopathy, with moderate (ETDRS score = 43) non-proliferative diabetic retinopathy (NPDR) without macular edema or with diabetic macular edema (DME) were enrolled. Patients’ records and blood samples were de-identified and numerically coded to protect patient privacy. Sera were isolated through centrifugation of whole blood collected in vacutainer serum separator tubes (BD SST#367985, Franklin Lakes, NJ, USA). Blood was also collected in vacutainer sodium heparin tubes (BD #236874 Franklin Lakes, NJ, USA) for isolation of peripheral blood mononuclear cells (PBMC). PBMC were negatively selected through magnetic bead separation using the RoboSep-S (Stemcell Technologies, Vancouver, BC, Canada) and the EasySep human PBMC isolation kit (Stemcell Technologies #19654, Vancouver, BC, Canada).

### 2.2. Quantitative PCR Analysis of STEAP4 Expression in PBMC of Diabetics

According to the manufacturer’s directions, RNA was extracted from PBMC using the RNeasy kit (Qiagen, Hilden, Germany). Samples with an OD260/280 ratio of 2.0 were used to generate cDNA using qScript (Quanta Bio, Beverly, MA, USA). Quantitative PCR was performed on a Lightcycler 96 System (Roche, Basel, Switzerland), using Fast Start Universal SYBR green (Roche, Basel, Switzerland) as the detecting probe. *STEAP4* expression was quantified using human *STEAP4* primers (NM_024636) and human *ACTB* primers (NM_001101) as the loading control. The 2^–ΔΔCt^ was equated to determine if *STEAP4* mRNA expression was upregulated in PBMC of diabetics with varying DR severity than non-diabetic controls. Graphed data is the fold increase of *STEAP4* in PBMC of diabetics without retinopathy, with NPDR, or DME compared to levels of *STEAP4* expression in PBMC of non-diabetic human controls.

### 2.3. Streptozotocin Induced Diabetes in C57BL/6 Mice

Male C57BL/6 mice (strain no. 000664, The Jackson Laboratory, Bar Harbor, ME, USA) received IP injections of 60 mg/Kg of BW (STZ) streptozotocin (MP Biomedicals, Irvine, CA, USA) in 0.1 M citrate buffer (pH 4.5) for five consecutive days; after a 6-h fast. Food was returned to the mice immediately after each injection, and water was provided ad lib. Diabetes was confirmed by a 6-h fasted blood glucose (FBG), with concentrations greater than 275 mg/dL. Diabetic conditions were verified by three separate FBG measures 14–21 days after the last STZ injection. Blood glucose was measured using a conventional consumer glucose testing meter and strips (FBG of non-diabetic mice were 150 ± 40 mg/dL). Diabetic conditions were further confirmed by quantifying the hemoglobin A_1C_ percentage using the Crystal Chem Mouse A_1C_ kit and Controls (Elk Grove Village, IL, USA). Mice were weighed weekly. When body weight loss exceeded 10% per week, 0–0.2 Units of insulin (Humulin N, NPH, Eli Lilly, Indianapolis, IN, USA) were administered on an as-needed basis to maintain body weight.

### 2.4. Anti-STEAP4 Neutralizing Antibodies and Treatment Regimen

The anti-STEAP4 neutralizing antibody is an antagonist that blocks Fe^3+^ binding to the STEAP4 C-terminal domain. Human STEAP4 neutralizing peptide was conjugated to STEAP4 antibody (PEP-0524-neutralizing peptide + PA5-20407-antibody, Thermo Fisher, Waltham, MA, USA) per manufacturer’s instructions and used to block STEAP4 activity in human Müller glia and human retina endothelial cells (hREC). Neutralizing peptide (CVDNTLTRIRQGWERN of NP_078912.2) was conjugated to STEAP4 antibody, per the manufacturer’s instructions (MyBioSource, San Diego, CA, USA) and used to inhibit STEAP4 activity in 661 W mouse cells and mouse retinas. For intravitreal injections, anti-STEAP4 was further conjugated to keyhole limpet hemocyanin (Imject mcKLH, Thermo Fisher, Waltham, MA, USA). Non-diabetic controls and diabetic C57BL/6 mice received one intravitreal injection of 1 μL phosphate buffered saline (PBS) containing 5 μg of KLH conjugated with anti-STEAP4 (MBS421017-neutralizing peptide + MBS426997-STEAP4 antibody); 1-week after diabetic conditions were confirmed.

Anti-STEAP4 was administered through intravitreal injections to anesthetized mice that received an IP injection of Ketamine: Xylazine cocktail. Proparacaine was applied to numb the eye, and tropicamide was applied to dilate the pupil. A beveled needle (34-gauge NanoFil, World Precision Instruments, Sarasota, FL, USA) was used to establish a route to the vitreous cavity. The needle was immediately replaced with a blunt-tip 34-gauge needle, attached to a micro-syringe (Sub-Microliter Injection System, and World Precision Instruments, Sarasota, FL, USA) filled with PBS containing 5 μg of anti-STEAP4. After anti-STEAP4 was administered through an intravitreal injection of one eye, the eyes were covered with GenTeal 0.3% Hypromellose gel (Alcon, Fort Worth, TX, USA) to protect the corneas from drying. To prevent infection, ophthalmic bacitracin-neomycin-polymyxin triple antibiotic ointment was applied to the procedure eye once daily for three consecutive days after the anti-STEAP4 injection was administered.

### 2.5. Western Immunoblot and Automated WES Analysis of Retina Lysates

For Western immunoblot analysis, retinas were pooled (*n* = 6) and homogenized in RIPA buffer (Thermo Fisher, Rockland, IL, USA). Levels of protein were quantified in each sample using a BCA assay (Pierce, Waltham, MA, USA) and normalized in the buffer to generate equal amounts of protein in each sample. Samples of protein lysates were loaded onto SDS Tris-glycine gels and transferred to a PVDF membrane using the BioRad Trans-blot Turbo system (BioRad, Hercules, CA, USA). Immunoblots were incubated in Intercept blocking buffer (Li-Cor, Lincoln, NE, USA) for 1 h at room temperature and further incubated in blocking buffer containing 1:1000 of STEAP4 antibody (ABS998, Millipore, Burlington, MA, USA) for 18 h at 4 °C. Immunoblots were washed, and incubated with the secondary antibody, and then imaged on a Li-Cor Odyssey Imaging System using studio 6.0 software (LiCor, Lincoln, NE, USA). Quantification was determined by normalizing samples to levels of β-actin in each sample (#8826, Abcam, Cambridge, MA, USA).

Alternatively, levels of Occludin in protein lysates were quantified using automated WES (Protein Simple, Biotechne, Minneapolis, MN, USA). For WES analysis, individual retinas were dissected from freshly collected mouse eyes and immersed in 30 μL of RIPA buffer containing protease inhibitor cocktail (Pierce Biotechnology, Rockford, IL, USA) and placed on ice. Samples were then homogenized in a bead homogenizer for 5 min. The resulting lysates were then centrifuged at 4 °C for 15 min. Collected supernatants were diluted 40:1 for protein concentration analysis using the Pierce BCA Protein Assay kit (A55864, Thermo Fisher, Waltham, MA, USA). Each sample was run in triplicate, and the readings were averaged to obtain a protein concentration for each retinal sample. Prior to WES analysis, each antibody and lysate sample were optimized per the manufacturer’s instructions. Normalized lysate samples containing 1.0 mg/mL of protein, the anti-mouse Occludin antibody (DSHB, Iowa City, IA, USA) diluted 1:10 in WES antibody diluent, and the anti-mouse β-actin antibody (#8226, Abcam, Waltham, MA, USA) diluted 1:10 in WES antibody diluent were loaded onto capillary gels in a WES cartridge; according to manufacturer’s instructions. Levels of Occludin and β-actin were quantified by a WES-generated electropherogram for dropped lines algorithmic analysis, using Compass for Simple Western v6.3 software (Protein Simple, Minneapolis, MN, USA). The area under the curve of the electropherogram was equated to quantitate the level of Occludin and β-actin in each sample of individual retinas from untreated or anti-STEAP4-treated diabetic mice.

### 2.6. Immunofluorescence and Microscopy Analysis of Retina Cross Sections

Slides of retina cryostat sections from non-diabetic and diabetic mice were blocked in 5% goat serum (R&D Systems Normal Goat Serum #DY005, Minneapolis, MN, USA) for 2 h at room temperature. For iron analysis, anti-Ferritin antibody (Abcam #ab75973, Cambridge, UK) was diluted 1:100 in PBS + 0.05% TWEEN-20 (Promega #H5152, Madison, WI, USA), applied to slides and incubated for 18 h at 4 °C. Slides were washed in 3× PBS + 0.05% TWEEN-20 and incubated with goat secondary antibody conjugated to Alexa Fluor 488 fluorochrome (Jackson Immuno Research Labs, West Grove, PA, USA) for 2 h at room temperature. Slides were mounted using DAPI-Fluor mount-G (Southern Biotech #0100-20, Birmingham, AL, USA). Alternatively, cross sections were incubated with primary antibodies; anti-STEAP4 and/or anti-Vimentin (anti-STEAP4 #ABS988, Millipore, Burlington, MA, USA or anti-Vimentin #ab92547, Abcam, Waltham, MA, USA) for 18 h at 4 °C. Slides were washed with PBS + 0.05% TWEEN-20 and then stained with secondary antibodies (Alexa Fluor 488 or 647, Jackson Immuno Research Labs, West Grove, PA, USA). Since both antibodies were generated from a rabbit host, rabbit serum and Fab fragments were used to prevent non-specific staining.

Lipid peroxidation was examined using 4-Hydroy-2-nonenal (4HNE) staining in retina cryostat sections of diabetic mice. Sections were blocked in 5% normal goat serum, stained with rabbit anti-serum directed against 4HNE (HNE11-S, Alpha Diagnostic, San Antonio, TX, USA), and anti-STEAP4 or anti-Vimentin. Slides were then incubated with Alexa 488 or 647 conjugated secondary antibodies. Images were then viewed on a Leica DMI 6000B widefield microscope and the Olympus Fluoview FV1200 Laser Scanning Confocal Microscope for 4HNE colocalization analyses. To quantify the levels of STEAP4 and Ferritin in the retina cross-sections, 14-bit images were captured using a Retiga EXi camera (Q-imaging, Austin, TX, USA). An identical threshold of the STEAP4 or Ferritin signal was created in MetaMorph 7.10.5 imaging software (Molecular Devices, Downingtown, PA, USA) and applied to all images. The entire retina was created as the threshold area in MetaMorph imaging software to equate an average intensity value of STEAP4 or Ferritin in the total retina. To calculate the level of STEAP4 in the photoreceptor and Müller glia layers, ten equal-sized regions were created in MetaMorph imaging software and overlaid at random on these specific layers of the retina. Fluorescent intensity values of STEAP4 in these threshold areas were quantified in Fluorescent Intensity Units (FIU) using MetaMorph imaging software.

### 2.7. Retina Cell Lines

Human retina endothelial cells (hREC) of the microvasculature were purchased from Cell Systems (Catalog #ACBRI, Kirkland, WA, USA). The purity of cells was verified by the Cell System to have cytoplasmic uptake of Di-I-Ac-LDL and positivity of cytoplasmic VWF/Factor VII and CD31. Cells were cultured in the manufacturer’s recommended media.

Human Müller glia was isolated from the posterior section of retinal globes from human cadaver eyes (Eversight, Cleveland, OH, USA). Müller glia was mechanically isolated, cultured in DMEM/HAM F12 media at 37 °C with 5% CO_2_, and collected after three passages. Cell purity was confirmed by flow cytometry analysis (>99% Vimentin^+^/GS^+^).

The 661 W cells were a generous gift from Dr. Timothy Kern. This photoreceptor-like cone cell line originated from mouse retina tumors of transgenic mice expressing Sv40T-antigen controlling the interphotoreceptor retinoid-binding protein promoter, which expresses photoreceptor cone opsins [22,23].

### 2.8. Detection of Reactive Oxygen Species

Human Müller glia or 661 W cells (1 × 10^5^ cells/well) were cultured in euglycemic or hyperglycemic conditioned media. All treated cells were cultured in optimal conditions (media containing 5 mM of glucose) with 1, 2, or 5 μg of anti-human or anti-mouse STEAP4 neutralizing antibody. Cells were incubated at 37 °C for 2 h. Untreated and anti-STEAP4 treated cells either remained in media containing 5 mM of glucose or the media was changed, and cells were cultured in hyperglycemic conditions (media containing 25 mM of glucose) for 18 h. Cells were collected and incubated with 10 μM H_2_DCFDA ((2′,7′-dichlorodihydrofluorescein diacetate) Invitrogen # D399, Carlsbad, CA, USA) at 37 °C for 30 min in the dark. H_2_DCFDA was used to quantify levels of ROS (green fluorescence) on a BD Accuri C6 flow cytometer (BD, San Jose, CA, USA).

Alternatively, retina cells were isolated as previously described [24]. Briefly, cells were isolated from retinas using the Worthington papain dissociation kit (Worthington Biochemical #LK003150, Lakewood, NJ, USA), followed by collagenase incubation for 1 h at 37 °C. Retina cells were washed in PBS and incubated in H_2_DCFDA for 30 min in the dark at 37 °C. Levels of ROS were quantified by measuring specific H_2_DCFDA green fluorescence (RLU × 10^3^) using a BD Accuri C6 flow cytometer.

### 2.9. Human Sera Induced Retinal Vascular Cell Death Analysis

Human vascular cell death assays were performed, as previously described [25]. Briefly, 1 × 10^6^ hREC/well were cultured in a 6-well plate containing 2 mL of Cell Systems’ media with or without 5 μg of anti-STEAP4 for 2 h at 37 °C with 5% CO_2_. After cells reached 80% confluency, 1 mL of media was removed and replaced with 1 mL of human serum of non-diabetic patients or diabetics with NPDR+DME. Cells were incubated with media containing sera for 18 h at 37 °C with 5% CO_2_. Cells were collected, and stained with 7-AAD (eBioscience #00-6993-50, San Diego, CA, USA) and analyzed for cell death by flow cytometry analyses of 7-AAD positivity.

## 3. Results

### 3.1. Clinical Data of Non-Diabetic and Diabetic Patient Donors

In concurrence with IRB #1588931 Protocol, clinical data and blood were collected from non-diabetic and Type II diabetic patients at Louis Stokes Cleveland VA Medical Center after written consent was provided by all enrolled donors. Patient samples were acquired from Caucasian (55%) and African American (45%) men, with an age range of 25–75 years old. Patients were enrolled during their annual eye exams. Donors were not diagnosed with any other retinal disease. The severity of retinopathy was determined by ETDRS scoring during their eye exams. Per the donors’ patient records, there were no significant differences in cholesterol levels amongst each group. NFBG (non-fasted blood glucose) was measured at the time of sample collection to affirm the non-diabetic or diabetic conditions of the donors. As shown in Table 1, NFBG levels were significantly higher in all diabetics than in non-diabetics (*n* = 15/group).

Hemoglobin A_1C_ percentages (HbA_1C_) of all mice and patient donors were used to affirm diabetic conditions. Because HbA_1C_ scores have less variation over an elongated period of time than NFBG and FBG scores. In both humans and mice, non-diabetics have HbA_1C_ scores below 5.7%, and diabetics have HbA_1C_ scores above 6.5% [26]. Patients with pre-diabetes have HbA_1C_ scores between 5.7–6.4%; any mouse or human donor that had HbA_1C_ scores in this range was excluded from this study. As shown in Table 1, all non-diabetic donors had HbA_1C_ scores below 5.7%, and all diabetic donors had HbA_1C_ scores above 6.5%. Thus, all diabetics had significantly higher HbA_1C_ scores than non-diabetics.

### 3.2. Clinical Data of Non-Diabetic and Diabetic Mice

Diabetes was induced in mice through 5 consecutive STZ injections. Diabetes in mice was confirmed 3-weeks after mice received the last STZ injection. FBG measures greater than 275 mg/dL confirmed diabetic conditions, while non-diabetic mice had FBG concentrations of 150 ± 40 mg/dL. To affirm diabetes at the time of harvest, HbA_1C_ scores were measured 2-months after the last FBG analysis. As shown in Table 2, all diabetic mice had A_1C_ scores above 6.5%, and all non-diabetic mice had A_1C_ scores below 5.7%. Thus, all diabetic mice had significantly higher HbA_1C_ scores than non-diabetic mice. However, there were no significant differences in HbA_1C_ scores of untreated and treated diabetic mice. Finally, STZ-diabetic mice normally have significantly lower body weights than non-diabetic mice [27]. As shown in Table 2, all diabetic mice had significantly lower body weights than all non-diabetic mice. However, there were no significant differences in body weights between the treated diabetic mice and the untreated diabetic mice (Table 2).

### 3.3. STEAP4 Expression Is Upregulated in PBMC of Diabetic Patients

**Figure 1:**
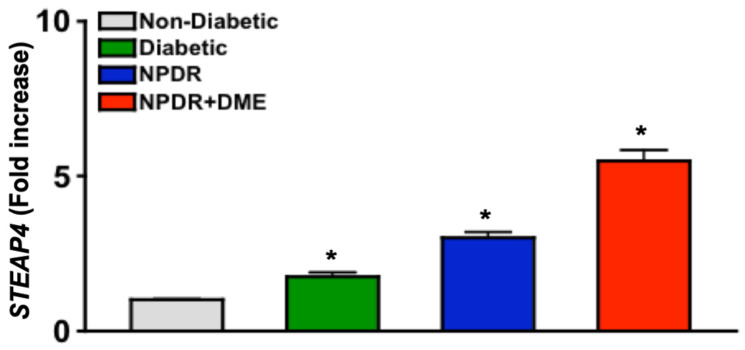
*STEAP4* expression in PBMC of non-diabetic and diabetic patients. Fold increase of *STEAP4* expression in PBMC (*n* = 15/group) from non-diabetic patients (grey), diabetics without retinopathy (Diabetic: green), and diabetics with non-proliferative diabetic retinopathy (NPDR: blue) without and with diabetic macular edema (NPDR + DME: red). Fold increases were calculated using 2^−ΔΔCt^, * = *p* < 0.01 was equated using 1-way nested ANOVA and unpaired student *t*-test.

The *STEAP4* gene is constitutively expressed in PBMC [28]. In rheumatoid arthritis and sepsis, upregulated *STEAP4* expression in PBMC initiated ROS production that caused pathologic oxidative stress [28,29]. To determine if *STEAP4* is upregulated in PBMC of human diabetics with diabetic retinopathy, mRNA was isolated from human PBMC (*n* = 15/group) for qPCR analyses of Ct scores, which were used to equate the *STEAP4* fold change using 2^−ΔΔCt^ calculations. As shown in Figure 1, *STEAP4* expression significantly increases in human PBMC in all diabetic donors. It is further notable that there is a gradient increase of *STEAP4* from low levels in non-diabetics to successively higher levels in diabetics with more severe diseases (Figure 1). The correlation of *STEAP4* to the severity of diabetic retinopathy suggests that STEAP4 could be a clinically relevant biomarker for the progression of diabetic retinopathy.

### 3.4. STEAP4 and Iron Is Significantly Increased in the Retinas of Diabetic Mice

**Figure 2:**
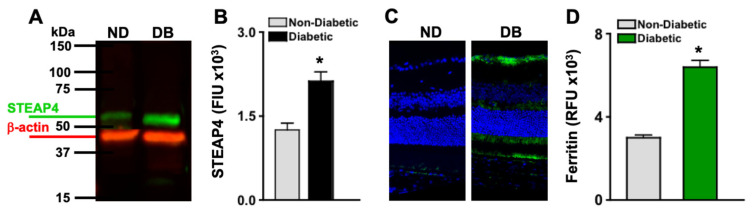
STEAP4 and iron in the retinas of non-diabetic and diabetic mice. (**A**) Representative immunoblot of STEAP4 (52 kDa: green) and β-actin (42 kDa: red) of protein lysates, and (**B**) FIU (fluorescent intensity) quantification of all protein samples (*n* = 3/group) from non-diabetic (grey) and diabetic (black) mice; 2-months post-diabetes. (**C**) Representative images of ferritin (green) and DAPI (blue) stained retina cross sections of non-diabetic (ND) and diabetic (DB) mice. (**D**) Graph of MetaMorph quantification of ferritin fluorescence in all retinas examined (*n* = 5) of non-diabetic (grey) and diabetic (green) mice; 2-months post diabetes. * = *p* < 0.01 per ANOVA and *t*-test.

STEAP4 protein is upregulated in retinal mitochondria in STZ-diabetic male rats [14]. To affirm that STEAP4 is upregulated in the retinas of diabetic mice, protein lysates of 6 pooled retinas per sample (*n* = 3/group) from non-diabetic and diabetic mice were examined by Western immunoblot; 2 months post-diabetes. As shown in Figure 2A, STEAP4 was detected in the retinal protein of both non-diabetic and diabetic mice. Per Licor quantification, STEAP4 is significantly increased in the retinas of diabetic mice than in non-diabetic mice (Figure 2B). STEAP4 is activated when iron is upregulated [30,31]. Per the literature, diabetes upregulates ferric iron in the serum, which induces ferrous iron uptake in the retina [18]. When ferrous iron accumulates in the retina, an iron storage protein known as ferritin is upregulated [12]. Thus, ferritin staining is used as an indicator of iron accumulation in cells, tissue, and the retina [12,32]. As shown in Figure 2C, only negligible levels of ferritin are detected in the retinas of non-diabetic mice. Yet, there are detectable levels of ferritin in the retinas of diabetic mice; 2 months post-diabetes. Per MetaMorph quantification, relative fluorescence units (RFU) of ferritin are significantly increased in the retinas of diabetic mice than in non-diabetic mice (Figure 2D). The results provide strong evidence that diabetes mediates the upregulation of STEAP4 and iron uptake in the murine retina.

### 3.5. STEAP4 Is Upregulated in the Photoreceptor and Müller Glia Layers in Retinas of Diabetic Mice

**Figure 3:**
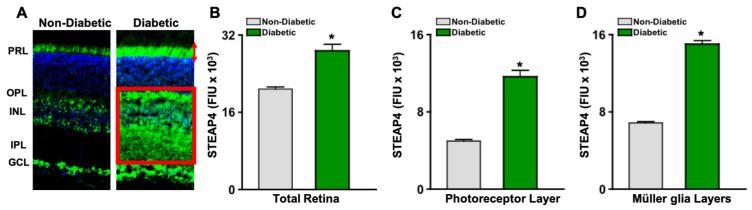
STEAP4 in photoreceptor and Müller glia layers of murine retinas. (**A**) Images of STEAP4 (green) and DAPI (blue) in 8 μM cross-sections of retinas from non-diabetic and diabetic mice. STEAP4 is outlined by red arrows in the photoreceptor layer and a red box in the Müller glia layers. Levels of STEAP4 fluorescence (FIU) in the total retina (**B**), photoreceptor layer (**C**), and retina layers where Müller glia reside (**D**) of non-diabetic (grey) and diabetic (green) mice (*n* = 5/group); 2-months post-diabetes. * = *p* < 0.01 per ANOVA and post-hoc unpaired student *t*-test.

To further determine where STEAP4 is upregulated in the retina, cross sections of retinas from non-diabetic and diabetic male mice were stained with anti-STEAP4 (green) and DAPI (blue) for microscopy analyses. As shown in Figure 3A, STEAP4 (green) was prevalently upregulated in the neural retina layers (PRL: red arrows, and INL stretching to IPL and OPL: red box) of the diabetic retina. Per Metamorph analyses of all retinas (*n* = 5/group), levels of STEAP4 are significantly increased in the entire retina of diabetic than in non-diabetic mice; 2-months post-diabetes (Figure 3B). Notably, levels of STEAP4 significantly increase in the layers of the retinas where photoreceptors (Figure 3C) and Müller glia (Figure 3D) reside in diabetic mice in comparison to non-diabetic mice.

### 3.6. STEAP4 and 4HNE Colocalize in Photoreceptors and Müller Glia of Diabetic Mice

**Figure 4:**
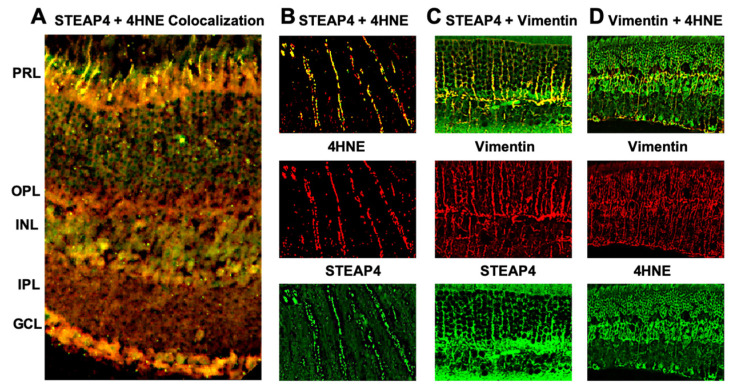
STEAP4 and 4HNE in photoreceptors and Müller glia of diabetic mice. (**A**) Widefield image of merged (yellow) STEAP4 (green) and 4HNE (red) in a retinal cross section from a diabetic mouse. (**B**) Representative confocal images of the photoreceptor outer segment, displaying STEAP4 (green) and 4HNE (red) colocalization (yellow). (**C**) Confocal images of STEAP4 (green) colocalized (yellow) in Vimentin (red) stained Müller glia. (**D**) Confocal images of 4HNE (green) colocalization (yellow) with Vimentin (red) stained Müller glia. All images are representative of retina cross-sections from diabetic mice analyzed (*n* = 5) 2-months after diabetic conditions were confirmed.

Diabetes initiates photoreceptor cells and Müller glia to produce reactive oxygen species (ROS), which enhances retinal oxidative stress [5,6]. To examine the impact of STEAP4 on retinal oxidative stress, lipid peroxidation (4-Hydroxy-2-nonenal (4HNE)) was examined in the retina of diabetic mice; 2-months post-diabetes. Retina cross-sections from diabetic mice were stained for STEAP4 and 4-HNE for microscopy analysis. As shown in Figure 4A, colocalization (yellow) of 4-HNE and STEAP4 was detected in the photoreceptor layer (PRL) and the inner nuclear layer (INL, where Müller glia resides). Confocal analysis confirmed colocalization (yellow) of STEAP4 (green) and 4-HNE (red) in photoreceptors (Figure 4B). Also, Vimentin (red) stained Müller glia was colocalized (yellow) with STEAP4 (green: Figure 4C) and 4HNE (green: Figure 4D). These results suggest that STEAP4 impacts oxidative stress in Müller glia and photoreceptors in the retinas of diabetic mice.

### 3.7. Anti-STEAP4 Halts ROS Production in Müller Glia and Photoreceptor Cells

**Figure 5:**
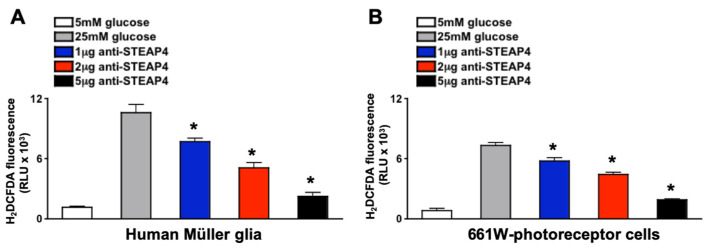
ROS production in anti-STEAP4 treated photoreceptors and Müller glia. Müller glia (**A**) (*n* = 6) and (**B**) 661 W photoreceptor-like cone cells (*n* = 6) were incubated in euglycemic conditioned media containing 5 mM of glucose (white) or hyperglycemic conditioned media containing 25 mM of glucose without (grey) or with: 1 μg (blue), 2 μg (red), or 5 μg (black) of anti-STEAP4 for 18 h. Human Müller glia (**A**) and mouse 661 W cells (**B**) were collected and incubated with H_2_DCFDA (ROS indicator) for flow cytometry quantification of ROS (H_2_DCFDA fluorescence (RLU × 10^3^)). * = *p* < 0.01, which was equated by 2-way ANOVA and Tukey’s post-hoc unpaired student *t*-test.

Neutralizing anti-STEAP4 antibody is an antagonist that binds to the C-terminal portion of STEAP4, causing a conformational change in the transmembrane binding groove of STEAP4. This inhibits the biological activity of STEAP4 and halts STEAP4-dependent ROS production [30]. To validate anti-STEAP4, 1 × 10^5^ Müller glia or 661 W photoreceptor-like cone cells were incubated with varying concentrations of anti-STEAP4 in euglycemic conditions (5 mM of glucose in media) for 2 h at 37 °C. Cells were then cultured in hyperglycemic conditions (25 mM of glucose in media) for 18 h at 37 °C, which initiates these cells to produce ROS [33,34,35]. Only negligible levels of ROS were detected in Müller glia (Figure 5A) and photoreceptors (Figure 5B) cultured in euglycemic conditions (media containing 5 mM of glucose). However, Müller glia and photoreceptors produced high levels of ROS when cultured in hyperglycemic conditions (25 mM of glucose). ROS production was significantly decreased when retina cells were cultured with anti-STEAP4 (αSTEAP4). A dose-dependent response to anti-STEAP4 was induced in both Müller glia (Figure 5A) and 661 W-photoreceptor cells (Figure 5B). ROS levels were inversely decreased when retina cells were treated with 1 μg, 2 μg, and 5 μg of anti-STEAP4. Similar (no significant difference) levels of ROS were produced by Müller glia and photoreceptor cells treated with 5 μg of anti-STEAP4 as cells cultured in optimal euglycemic conditions. These results provide evidence that STEAP4 induces Müller glia and photoreceptors to produce ROS under hyperglycemic conditions. While elucidating that 5 μg of anti-STEAP4 is sufficient to halt hyperglycemia-mediated ROS.

### 3.8. Anti-STEAP4 Treatment Halts Retinal Oxidative Stress in Diabetic Mice

**Figure 6:**
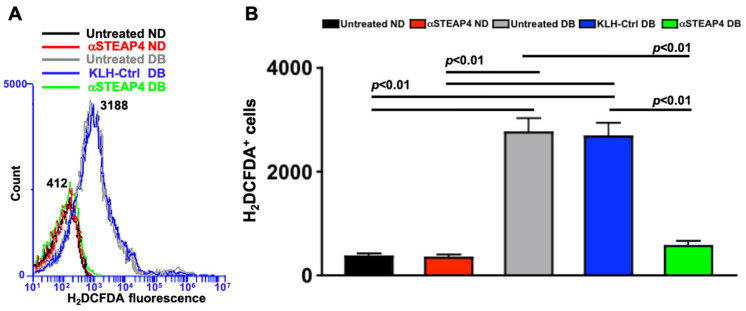
ROS in anti-STEAP4 treated murine retinas. (**A**) Representative histogram overlay from flow cytometry analysis of H_2_DCFDA^+^ (ROS producing) retina cells from untreated non-diabetic (black: with a peak value of 412), anti-STEAP4 treated non-diabetic (red), untreated diabetic (grey: with a peak value of 3188), diabetic KLH control (blue), and anti-STEAP4 treated diabetic (green) C57BL/6 mice; 2-months post-diabetes. (**B**) Quantification of H_2_DCFDA^+^ (ROS producing) retina cells in all samples (*n* = 5/group) analyzed by flow cytometry; *p*-values were equated using 2-way ANOVA and Tukey’s post-hoc unpaired student *t*-tests.

To further ascertain a role for STEAP4 in diabetes-mediated retinal oxidative stress, non-diabetic (ND) and diabetic (DB) C57BL/6 mice received one intravitreal injection of 5 μg of anti-STEAP4 1-week after diabetes was confirmed. Retinas were collected, processed, and incubated with H_2_DCFDA to quantify levels of ROS in retina cells of individual retinas by flow cytometry analysis; 2-months post-diabetes. Per the flow cytometry histogram overlay (Figure 6A) and the fluorescent quantification of H_2_DCFDA^+^ cells (Figure 6B), there was no difference in the level of ROS in the retinas of untreated (black) than anti-STEAP4 treated (red) non-diabetic mice. Levels of ROS were significantly higher in the retinas of untreated (grey) and KLH-treated (carrier protein KLH-control: blue) diabetic mice compared to non-diabetic mice. Meanwhile, diabetes-mediated ROS was significantly decreased in the retinas of anti-STEAP4 treated (green) diabetic mice when compared to the untreated and KLH-treated diabetic mice. Notably, no significant differences in the levels of ROS were detected in the anti-STEAP4-treated diabetic mice and the non-diabetic controls. These data provide strong evidence that STEAP4 impacts oxidative stress in diabetic retinas.

### 3.9. Anti-STEAP4 Treatment Impedes Diabetes-Mediated Occludin Degradation in Murine Retinas

**Figure 7:**
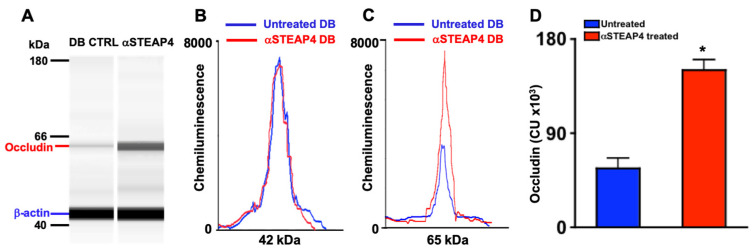
Levels of Occludin in lysates from murine retinal vasculature. (**A**) Representative WES gel of Occludin (65 kDa: red) and β-actin (42 kDa: blue). Representative electropherograms of β-actin (**B**) and Occludin (**C**) of protein lysates from retinal vasculature of untreated (DB CTRL: blue) and anti-STEAP4 treated (αSTEAP: red) diabetic mice. (**D**) Levels of Occludin in all protein lysate samples (*n* = 5/group) analyzed from untreated (blue) and anti-STEAP4 treated (red) diabetic mice; 2-months post-diabetes. * = *p* < 0.01; per 2-way ANOVA and post-hoc unpaired student *t*-test.

Tight junction protein degradation is the earliest vascular impairment detectable in diabetic mice [36]. Hyperglycemia impairs the expression of Occludin (a tight junction protein) in the retinal vasculature during the onset of diabetic retinopathy [36]. To define the impact of STEAP4 on Occludin degradation, diabetic mice remained untreated or received one intravitreal injection of PBS containing 5 μg of anti-STEAP4; 1-week post-diabetes. Levels of Occludin were examined in protein lysates of retina vasculature of untreated and anti-STEAP4 treated diabetic mice by automated Western immunoblot (WES) analysis. Comparable levels of protein in each sample were examined and confirmed per the β-actin (42 kDa) loading control (Figure 7A,B). Levels of Occludin (65 kDa) were higher in the lysates of the retinal vasculature from anti-STEAP4 treated than untreated diabetic mice (Figure 7A,C). Occludin was significantly increased in all protein lysate samples from the retinal vasculature of anti-STEAP4 treated diabetic mice than untreated diabetic mice (*n* = 5/group); 2-months post-diabetes (Figure 7D). These results provide strong evidence that STEAP4 impacts the integrity of the retinal vasculature in diabetic mice.

### 3.10. Anti-STEAP4 Inhibits Diabetes-Mediated Human Retina Endothelial Cell Death

**Figure 8:**
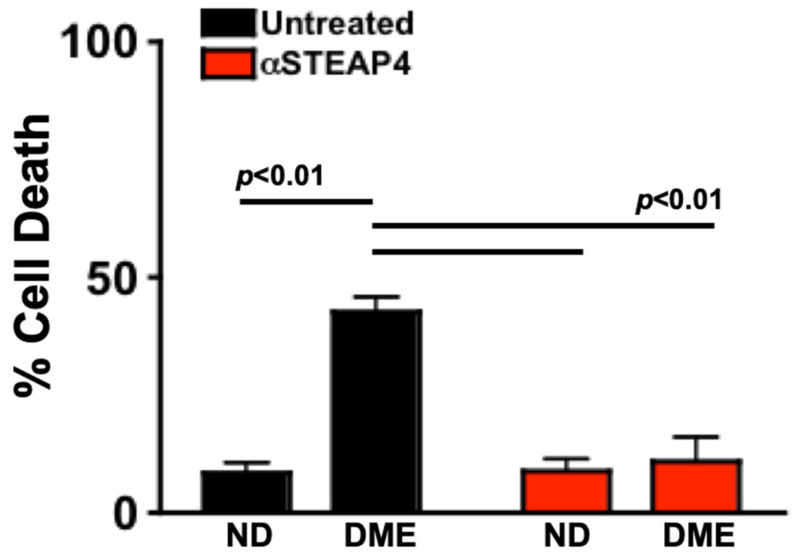
Human retina endothelial cell death in cells cultured with patient sera. Untreated (black) or anti-STEAP4 treated (red) hREC (1 × 10^6^) were cultured with sera of non-diabetics (ND) or sera of patients with NPDR and diabetic macular edema (DME) for 18 h. Cells were collected and incubated with 7-AAD for flow cytometry analysis of hREC cell death (*n* = 15/group). The *p*-value was calculated using 2-way ANOVA analysis and Tukey’s post-hoc unpaired student *t*-tests.

Retina endothelial cell (REC) death can be induced ex vivo when RECs are cultured with cytokines, sera, or immune cells of diabetic donors [25,37,38]. This proof of concept assay models the interactions that occur between endothelial cells in the vasculature of the retina and inflammatory processes circulating through the retinal vasculature. To determine if STEAP4 impacts human retina endothelial cell (hREC) death, untreated or anti-STEAP4 treated hREC were incubated with sera of non-diabetics (ND; *n* = 15) or sera of patients with diabetic macular edema (DME; *n* = 15) for 18 h at 37 °C with 5% CO_2_. Cells were collected and stained with 7-AAD for flow cytometry analysis of cellular death. As shown in Figure 8, retina endothelial cell death significantly increased when untreated hREC (black) were cultured with sera of patients with DME rather than sera of non-diabetics. Conversely, cell death was significantly decreased when hREC were treated with anti-STEAP4 (red) prior to being cultured with sera of patients with DME. These findings provide proof of concept that diabetes-mediated STEAP4 impacts the integrity of the retinal vasculature in patients with diabetic macular edema.

## 4. Discussion

The results of this study provide strong evidence that STEAP4 is upregulated in diabetic humans and mice. In the diabetic retina, both iron and STEAP4 are increased, which enhances retinal oxidative stress. This then initiates tight junction protein degradation and endothelial cell death in the diabetic retina. These subtle vascular impairments can lead to the development of diabetic retinopathy and vision loss [13]. When retina cells and mice were treated with anti-STEAP4, these intrinsic pathologies of diabetic retinopathy were halted.

STEAP 1-4 isoforms are metalloreductases that promote and regulate cellular uptake of iron to maintain homeostasis. All of these STEAP isoforms are expressed in the retina, but only STEAP3 regulates iron homeostasis in the thriving retina [18,19]. However, STEAP4 is the only isoform upregulated in the diabetic retina [16]. In murine models of colon cancer, systemic ablation of *Steap4* halted iron overload and disease pathogenesis in mice [18,19]. Analogous to these findings, we discovered both iron and STEAP4 were upregulated in the diabetic retina and that STEAP4 impelled photoreceptor cells and Müller glia to produce ROS. Thus, STEAP4 enhances retinal oxidative stress, which is a pivotal precursor to retinal pathogenesis and the development of diabetic retinopathy [5,6,7,8,9].

The etiology of diabetic retinopathy is multifactorial. Yet, multiple studies provide strong evidence that oxidative stress leads to the development of diabetic retinopathy [5]. Diabetes mediates chronic inflammation and oxidative stress, which causes gradual changes in the retinal microvasculature. These early-stage changes cause tight junction protein degradation and retina endothelial cell death [36]. Leading to vascular leakage, capillary non-perfusion, and the onset of non-proliferative diabetic retinopathy (NPDR). In some cases, these intrinsic pathologies mediate macular edema in the diabetic retina, altering vision [3]. Here, we discovered that *STEAP4* was significantly increased in PBMC of diabetics with NPDR and edema than in patients with less severe NPDR and no edema. Subsequently, vascular cell death was halted when human retina endothelial cells were treated with anti-STEAP4 prior to the addition of sera from patients with NPDR and edema. Providing strong evidence that STEAP4 impacts the pathogenesis of retinopathy by exacerbating oxidative stress in the diabetic retina.

There is lacking consensus on the role of STEAP4 in adipocytes and diabetes onset [32,39,40,41,42,43]. Opposing results of the impact of STEAP4 on insulin resistance and glucose intolerance in humans compared to mice have been reported [39,43]. The mechanistic basis for these reported inhibitory and inductive roles of STEAP4 in diabetes onset remains unclear. Defining the impact of STEAP4 in diabetes onset rather than its role in the onset of a diabetic complication (diabetic retinopathy) is beyond the scope of this study. Since there have been reported discrepancies between human results and Type II diabetic mice, we used the STZ-Type I diabetic mouse model to mechanistically examine our clinical findings. Thus, defining the role of STEAP4 in the development of diabetic retinopathy in STZ-diabetic mice removes the extrinsic factors that could impede the onset of diabetes, thus halting the onset of diabetic retinopathy in wild-type diabetic mice.

Collectively, the results from the human ex vivo and murine in vivo studies reveal the relevance of our original discovery that levels of *STEAP4* significantly increase in correlation to the severity of diabetic retinopathy. Thus, STEAP4 could be a clinically relevant biomarker for the progression of diabetic retinopathy and a novel therapeutic target for the development of diabetic retinopathy.

## 5. Conclusions

In conclusion, diabetes initiates *STEAP4* upregulation in PBMC that circulates through the retinal vasculature, iron (Fe^3+^) uptake in the retina, and increased STEAP4 in Müller glia and photoreceptors in the retina. In the retina, Fe^3+^ binds to the C-terminal transmembrane domain of STEAP4, which induces Müller glia and photoreceptors to produce ROS. This enhances oxidative stress in the retina, which leads to tight junction protein degradation and retina endothelial cell death. Meanwhile, the STEAP4 expression of PBMC further enhances endothelial cell death in the retinal vasculature. These intrinsic pathologies of vascular impairment can lead to the progression of diabetic retinopathy. Thus, STEAP4 could be a potentially novel therapeutic target and/or a clinically relevant biomarker for the development of diabetic retinopathy.

## Figures and Tables

**Table 1:** Clinical Data of Non-Diabetic and Diabetic Patient Donors.

Group	HbA_1C_ (%)	Cholesterol (mg/dL)	NFBG (mg/dL)
Non-Diabetic	5.53 ± 0.12	135.08 ± 22.61	90.27 ± 13.54
Diabetic without Retinopathy	7.78 ± 0.85 *	136.53 ± 23.83	203.33 ± 23.83 *
NPDR	7.59 ± 1.49 *	130.07 ± 21.72	201.27 ± 24.13 *
NPDR + DME	9.39 ± 1.89 *	135.21 ± 20.21	224.07 ± 22.79 *

* *p* < 0.01; per 2-way ANOVA and post-hoc unpaired student *t*-test analysis. Data are mean ± SD.

**Table 2:** Clinical data of non-diabetic and STZ-diabetic mice receiving anti-STEAP4 treatment.

Group	HbA_1C_ (%)	Body Weight (g)
Untreated Non-Diabetic	3.91 ± 0.26	35.22 ± 4.18
Untreated Diabetic	10.51 ± 2.02 *	27.56 ± 2.13 *
αSTEAP4-Treated Non-Diabetic	3.82 ± 0.25	36.01 ± 4.42
αSTEAP4-Treated Diabetic	11.05 ± 1.55 *	26.51 ± 1.01 *
KLH-Treated Non-Diabetic	3.89 ± 0.29	35.98 ± 4.07
KLH-Treated Diabetic	10.85 ± 1.11 *	27.09 ± 2.17 *

* *p* < 0.01 of diabetic compared to non-diabetic per treatment group. Data are mean ± SD.

## Data Availability

The datasets generated during and/or analyzed during the current study are available from the corresponding author upon reasonable request.

## References

[1] Saeedi P., Petersohn L., Salpea P., Malanda B., Karuranga S., Unwin N., Colagiuri S., Guariguata L., Motala A.M., Ogurtsova K. (2019). Global and regional diabetes prevalence estimates for 2019 and projections for 2030 and 2045: Results from the International Diabetes Federation Diabetes Atlas. Diabetes Res. Clin. Pract..

[2] Bloomfield H.E., Greer N., Newman D., MacDonald R., Carlyle M., Fitzgerald P., Rutks I., Wilt T.J. (2012). Predictors and Consequences of Severe Hyperglycemia in Adults with Diabetes—A Systemic Review of the Evidence.

[3] Duh E.J., Sun J.K., Stitt A.W. (2017). Diabetic retinopathy: Current understanding, mechanisms, and treatment strategies. JCI Insight.

[4] Zhao Y., Singh R.P. (2018). The role of anti-vascular endothelial growth factor (anti-VEGF) in the management of proliferative diabetic retinopathy. Drugs Context.

[5] Kowluru R.A., Chan P.S. (2007). Oxidative stress and diabetic retinopathy. Exp. Diabetes Res..

[6] Du Y., Veenstra A., Palczewski K., Kern T.S. (2013). Photoreceptor cells are major contributors to diabetes-induced oxidative stress and local inflammation in the retina. Proc. Natl. Acad. Sci. USA.

[7] Doganay S., Evereklioglu C., Er H., Türköz Y., Sevinc A., Mehmet N., Savli H. (2002). Comparison of serum NO, TNF-alpha, IL-1beta, sIL-2R, IL-6 and IL-8 levels with grades of retinopathy in patients with diabetes mellitus. Eye.

[8] Didion S.P. (2017). Cellular and oxidative mechanisms associated with interleukin-6 signaling in the vasculature. Int. J. Mol. Sci..

[9] Howell S.J., Lee C.A., Batoki J.C., Zapadka T.E., Lindstrom S.I., Taylor B.E., Taylor P.R. (2021). Retinal inflammation, oxidative stress, and vascular impairment is ablated in diabetic mice receiving XMD8-92 treatment. Front. Pharmacol..

[10] Chaudhary K., Promsote W., Ananth S., Veeranan-Karmegam R., Tawfik A., Arjunan P., Martin P., Smith S.B., Thangaraju M., Kisselev O. (2018). Iron overload accelerates the progression of diabetic retinopathy in association with increased retinal renin expression. Sci. Rep..

[11] Chen Y.J., Chen J.T., Tai M.C., Liang C.M., Chen Y.Y., Chen W.L. (2020). Serum iron and risk of diabetic retinopathy. Nutrients.

[12] Loh A., Hadziahmetovic M., Dunaief J.L. (2008). Iron homeostasis and eye disease. Biochim. Biophys. Acta.

[13] Hammes H.P., Feng Y., Pfister F., Brownlee M. (2011). Diabetic retinopathy: Targeting vasoregression. Diabetes.

[14] Wang W., Lo A.C.Y. (2018). Diabetic retinopathy: Pathophysiology and treatments. Int. J. Mol. Sci..

[15] Sinclair S.H., Schwartz S.S. (2019). Diabetic retinopathy-An underdiagnosed and undertreated inflammatory, neuro-vascular complication of diabetes. Front. Endocrinol..

[16] Wang J.J., Park K.S., Dhimal N., Shen S., Tang X., Qu J., Zhang S.X. (2022). Proteomic analysis of retinal mitochondria-associated ER membrane identified novel proteins of retinal degeneration on long-term diabetes. Cells.

[17] Jin Y., Wang L., Qu S., Sheng X., Kristian A., Maelandsmo G.M., Pällmann N., Yuca E., Tekedereli I., Gorgulu K. (2015). STAMP2 increases oxidative stress and is critical for prostate cancer. EMBO Mol. Med..

[18] Xue X., Bredell B.X., Anderson E.R., Martin A., Mays C., Nagao-Kitamoto H., Huang S., Györffy B., Grrenson J.K., Hardiman K. (2017). Quantitative proteomics identifies STEAP4 as a critical regulator of mitochondrial dysfunction linking inflammation and colon cancer. Proc. Natl. Acad. Sci. USA.

[19] Liao Y., Zhao J., Bulek K., Tang F., Chen X., Cai G., Jia S., Fox P.L., Huang E., Pizarro T.T. (2020). Inflammation mobilizes copper metabolism to promote colon tumorigenesis via an IL-17-STEAP4-XIAP axis. Nat. Commun..

[20] Pihlstrom N., Jin Y., Nenseth Z., Kuzu O.F., Saatcioglu F. (2021). STAMP2 expression mediated by cytokines attenuates their growth-limiting effects in prostate cancer cells. Cancers.

[21] Oosterheert W., van Bezouwen L.S., Rodenburg R.N.P., Granneman J., Förster F., Mattevi A., Gros P. (2018). Cryo-EM structures of human STEAP4 reveal mechanism of iron (III) reduction. Nat. Commun..

[22] Al-Ubaidi M.R., Font R.L., Quiambao A.B., Keener M.J., Liou G.I., Overbeek P.A., Baehr W. (1992). Bilateral retinal and brain tumors in transgenic mice expressing simian virus 40 large T antigen under control of the human interphotoreceptor retinoid-binding protein promoter. J. Cell Biol..

[23] Tan E., Ding X.Q., Saadi A., Agarwal N., Naash M.I., Al-Ubaidi M.R. (2010). Expression of cone-photoreceptor-specific antigens in a cell line derived from retinal tumors in transgenic mice. Investig. Ophthalmol. Vis. Sci..

[24] Sigurdardottir S., Zapadka T.E., Lindstrom S.I., Liu H., Taylor B.E., Lee C.A., Kern T.S., Taylor P.R. (2019). Diabetes-mediated IL-17A enhances retinal inflammation, oxidative stress, and vascular permeability. Cell Immunol..

[25] Lindstrom S.I., Sigurdardottir S., Zapaka T.E., Tang J., Liu H., Taylor B.E., Smith D.G., Lee C.A., DeAngelis J., Kern T.S. (2019). Diabetes induces IL-17A-Act1-FADD-dependent retinal endothelial cell death and capillary degeneration. J. Diabetes Complicat..

[26] Zhang X., Gregg E.W., Williamson D.F., Barker L.E., Thomas W., Bullard K.M., Imperatore G., Williams D.E., Albright A.L. (2010). A1C level and future risk of diabetes: A systemic review. Diabetes Care.

[27] Kern T.S., Tang J., Berkowitz B.A. (2010). Validation of structural and functional lesions of diabetic retinopathy in mice. Mol. Vis..

[28] Jiang H., Dong Y., Yan D., Wu Y., Wang Y., Ren Y., Mao G., Liang G., Liu W., Zhou Y. (2021). The expression of STEAP4 in peripheral blood predicts the outcome of septic patients. Ann. Transl. Med..

[29] Li W., Yin X., Yan Y., Liu C., Li G. (2021). STEAP4 knockdown inhibits the proliferation of prostate cancer cells by activating the cGMP-PKG pathway under lipopolysaccharide-induced inflammatory microenvironment. Int. Immunopharmacol..

[30] Zhou J., Ye S., Fujiwara T., Manolagas S.C., Zhao H. (2013). Steap4 plays a critical role in osteoclastogenesis in vitro by regulating cellular iron/reactive oxygen species (ROS) levels and cAMP response element-binding protein (CREB) activation. J. Biol. Chem..

[31] Giannou A.D., Lücke J., Kleinschmidt D., Shiri A.M., Steglich B., Nawrocki M., Zhang T., Zazara D.E., Kempski J., Zhao L. (2022). A critical role of the IL-22-IL-22 binding protein axis in hepatocellular carcinoma. Cancers.

[32] Catalan V., Gomez-Ambrosi J., Rodriguez A., Ramirez B., Rotellar F., Valenti V., Silva C., Gil M.J., Salvador J., Frühbeck G. (2013). Six-transmembrane epithelial antigen of prostate 4 and neutrophil gelatinase-associated lipocalin expression in visceral adipose tissue is related to iron status and inflammation in human obesity. Eur. J. Nutr..

[33] Albert-Garay J.S., Riesgo-Escovar J.F., Salceda R. (2022). High glucose concentrations induce oxidative stress by inhibiting Nrf2 expression in rat Muller retinal cells in vitro. Sci. Rep..

[34] Du Y., Miller C.M., Kern T.S. (2003). Hyperglycemia increases mitochondrial superoxide in retina and retinal cells. Free Radic. Biol. Med..

[35] Zhang T., Zhu L., Madigan M.C., Liu W., Shen W., Cherepanoff S., Zhou F., Zeng S., Du J., Gillies M.C. (2019). Human macular Muller cells rely more on serine biosynthesis to combat oxidative stress than those from the periphery. eLife.

[36] Barber A.J., Antonetti D.A., Gardner T.W. (2000). Altered expression of retinal occludin and glial fibrillary acidic protein in experimental diabetes. The Penn State Retina Research Group. Investig. Ophthalmol. Vis. Sci..

[37] Liu H., Tang J., Du Y., Saadane A., Samuels I., Veenstra A., Kiser J., Palczewski K., Kern T.S. (2019). Transducin 1, phototransduction and the development of early diabetic retinopathy. Investig. Ophthalmol. Vis. Sci..

[38] Veenstra A., Liu H., Lee C.A., Du Y., Tang J., Kern T.S. (2015). Diabetic Retinopathy: Retina-Specific Methods for Maintenance of Diabetic Rodents and Evaluation of Vascular Histopathology and Molecular Abnormalities. Curr. Protoc. Mouse Biol..

[39] Arner P., Stenson B.M., Dungmer E., Näslund E., Hoffstedt J., Ryden M., Dahlman I. (2008). Expression of six transmembrane protein of prostate 2 (STAMP2) in human adipose tissue associates with adiposity and insulin resistance. J. Clin. Endocrinol. Metab..

[40] Shayo S.C., Ogiso K., Kawade S., Hashiguchi H., Deguchi T., Nishio Y. (2022). Dietary obesity and glycemic excursions cause a parallel increase in STEAP4 and pro-inflammatory gene expression in murine PBMCs. Diabetol. Int..

[41] Sharma P.R., Mackey A.J., Dejene E.A., Ramadan J.W., Langefeld C.D., Palmer N.D., Taylor K.D., Wagenknecht L.E., Watanabe R.M., Rich S.S. (2015). An islet-targeted genome-wide association scan identifies novel genes implicated in cytokine-mediated islet stress in Type 2 diabetes. Endocrinology.

[42] Wellen K.E., Fucho R., Gregor M.F., Furuhashi M., Morgan C., Lindstad T., Valliancourt E., Gorgun C.Z., Saatcioglu F., Hotamisligil G.S. (2007). Coordinated regulation of nutrient and inflammatory responses by STAMP2 is essential for metabolic homeostasis. Cell.

[43] Chuang C.T., Guh J.Y., Lu C.Y., Wang Y.T., Chen H.C., Chuang L.Y. (2015). Steap4 attenuates glucose and S100B-induced effects in mesangial cells. J. Cell Mol. Med..

